# The association between bedtime smartphone use and anxiety symptoms: a network analysis of Chinese residents

**DOI:** 10.1186/s12888-025-06961-7

**Published:** 2025-05-26

**Authors:** Zheng Tian, Junshuai Lu, Yimiao Li, Nan Zhang, Yong Liu, Yibo Wu, Lan Wang

**Affiliations:** 1 School of Nursing, Tianjin Medical University, 22 Weitai Road, Heping District, Tianjin, 300070 China; 2 Tianjin Medical University, Tianjin Medical University Cancer Institute & Hospital, National Clinical Research Center for Cancer, Tianjin’s Clinical Research Center for Cancer, Tianjin, 300060 China; 3 School of Public Health, Peking University, Beijing, 100191 China

**Keywords:** Anxiety, Bedtime smartphone use, Problematic internet use, Network analysis

## Abstract

**Background:**

Bedtime smartphone use has become a common practice among modern individuals. The mechanisms underlying the association between bedtime smartphone use and anxiety are not fully understood in the whole population and across genders. Additionally, it remains unclear whether reducing problematic internet use (PIU) can lessen the association between bedtime smartphone use and anxiety.

**Methods:**

30,504 subjects were recruited from the Psychology and Behavior Investigation of Chinese Residents (PBICR). Logistic regression models were used to analyze the association between bedtime smartphone use and the risk of developing anxiety, as well as the interaction effect of problematic internet use (PIU) on this association. Multiple linear regression models were conducted to analyze the association between bedtime smartphone use and the severity of anxiety symptoms. Network analysis was utilized to identify core symptoms and to distinguish gender differences in the association between bedtime smartphone use and anxiety symptoms.

**Results:**

Compared to participants who used their smartphones for one hour or less before bedtime, using smartphones for more than one hour before bedtime was associated with a 9.1% higher likelihood of experiencing anxiety (OR = 1.091). The duration of bedtime smartphone use was positively correlated with anxiety severity ($\:\beta\:=$0.116, *P* < 0.001). In the network of bedtime smartphone use and anxiety symptoms in the general population, “Inability to stop or control worrying (GAD2)” and “Worrying too much about a variety of things (GAD3)” exhibited the highest centrality. The path coefficient between the duration of bedtime smartphone use and “Becoming annoyed or easily irritated (GAD6)” was the largest. Compared to males, the centrality of “Difficulty relaxing (GAD4)” was higher in females, and the path coefficients between the “The duration of mobile phone use before bedtime (phone)” and “Feeling nervous, anxious, or on edge (GAD1)”, “Inability to sit still due to restlessness (GAD5)”, and “Becoming annoyed or easily irritated (GAD6)” were greater in females. The centrality of “Feeling scared because something terrible seems to be about to happen (GAD7)” was higher in males. Individuals who reported both bedtime smartphone use of more than 1 h and PIU were associated with a 276.2% higher likelihood of experiencing anxiety. Those who reported bedtime smartphone use of more than 1 h and did not have PIU were associated with a 35.3% lower likelihood of experiencing anxiety (OR = 0.647).

**Conclusion:**

Using smartphones before bedtime was associated with a higher likelihood of experiencing anxiety and was positively correlated with the severity of anxiety in the general population. Worry symptoms showed the strongest association with bedtime smartphone use. The association between bedtime smartphone use and anxiety symptoms was stronger in women than in men. Using smartphones before bedtime for more than 1 h combined with PIU was associated with a higher likelihood of experiencing anxiety, while using a smartphone before bedtime for more than 1 h without PIU was associated with a lower likelihood of experiencing anxiety.

**Supplementary Information:**

The online version contains supplementary material available at 10.1186/s12888-025-06961-7.

## Introduction

With the rapid development of network technology and the dawn of the intelligent age, smartphones have become increasingly prevalent, rising to the status of an indispensable necessity in many people’s lives. With smartphones owned by 71% of the global population [1] and 104.7 million smartphone internet users in China alone [2]. A survey found that more than 80% of Chinese respondents use smartphones before bedtime [3]. Notably, bedtime smartphone usage has emerged as a widespread behavior pattern in China.

While the proliferation of smartphones has accelerated all areas of society and shortened social distances, using smartphones before bedtime has been associated with a higher risk of anxiety among adolescents [4, 5, 6]. Anxiety itself can exacerbate the risk of mortality and significantly affect interpersonal relationships and physical health [7, 8, 9]. However, the association between the duration of bedtime smartphone use and anxiety symptoms remains inadequately explored across the general population. Currently, there are multiple possible explanations for the detrimental impact of using smartphones before bedtime on anxiety. The Stimulus-Organism-Response (SOR) theory suggests that stimuli in the environment can trigger changes in an individual’s internal state, which then elicits emotional responses. Based on this theory, scholars believe that factors such as information overload, privacy invasion, and the information cocoon effect brought about by the use of digital technologies like smartphones, as environmental stimuli, can alter an individual’s psychological state, leading to anxiety and other negative emotions [10–11]. In particular, the blue light radiation emitted by smartphones when used before bed, as an environmental stimulus, has been shown to affect the user’s physiological state by suppressing the secretion of melatonin, continuously activating the sympathetic nervous system, and disrupting sleep structure, thereby exacerbating anxiety [12–13]. Moreover, the Social Upward Comparison Theory posits that individuals tend to compare themselves with others in certain aspects when using digital technologies, and this upward comparison typically triggers anxiety, inferiority, and other negative emotions. Therefore, when users browse social media on their phones before bed, they may experience anxiety due to the comparison impulses triggered by seeing the lives or achievements of others [14]. At the same time, the Social Replacement Hypothesis suggests that excessive use of smartphones, leading to immersion in the virtual world and a lack of face-to-face communication and social support in reality, limits the development of traditional interpersonal relationships and social skills, which in turn can lead to alienation from others and exacerbate anxiety [15, 16, 17]. Although there is currently no longitudinal study systematically investigating the impact of the time spent using smartphones before bed on anxiety, existing research has shown that staying up late and sleep deprivation caused by the blue light emitted by smartphones increase the risk of stress, depression, and other psychopathological conditions [18–19]. Given the constant rise in smartphone users across all age groups [1], the association between bedtime smartphone use and anxiety symptoms is likely a common concern. Therefore, it is necessary to explore this association in more detail across all age groups to provide a basis for developing early anxiety management strategies.

It is worth noting that existing studies have shown that anxiety sensitivity exhibits gender differences, as fluctuations in sex hormones (such as estradiol and progesterone) affect how individuals of different genders perceive and respond to external stimuli, which in turn influences the onset and manifestation of anxiety [20–21]. Therefore, compared to men, women may be more sensitive to the emotional impacts of behavior and are more prone to experiencing anxiety [21–22]. Research shows that women are more likely than men to use social media on smartphones more frequently. While women often face greater social media pressure, they have fewer coping mechanisms, making them more susceptible to anxiety from smartphone use [23]. Additionally, studies indicate that women are more vulnerable to the negative effects of bedtime smartphone use on sleep quality, which may increase their likelihood of experiencing anxiety due to smartphone use before bed [24–25]. Although pre-sleep smartphone use has been shown to be associated with an increased risk of anxiety, the differences in the relationship between smartphone use before sleep and anxiety symptoms across genders have not been fully explored. The gender differences in anxiety susceptibility may result in different anxiety experiences when individuals of different genders use smartphones before bedtime. Focusing on the potential relationship between pre-sleep smartphone use and anxiety symptoms in different gender groups is important for a deeper understanding of the underlying mechanisms and for formulating targeted strategies.

Previous studies have often treated the duration of bedtime smartphone use and anxiety as independent factors, without fully exploring the underlying mechanisms and their relationship [4, 5, 6]. This lack of comprehensive analysis has left the specific association between bedtime smartphone use and anxiety symptoms inadequately explored. Network analysis offers a valuable method to address this gap. By conceptualizing anxiety as a result of interacting symptoms like nervousness, difficulty relaxing, etc [26]., this approach can visualize how these variables influence each other. It reveals detailed relationships between factors, helping identify the most effective targets for intervention [27]. Therefore, identifying the core symptoms of anxiety in the network of bedtime smartphone use is crucial for formulating effective intervention strategies.

Problematic internet use (PIU) refers to the compulsive use of the internet for excessive amounts of time, leading to negative consequences in a person’s work, studies, and social life [28]. The widespread adoption of the internet has resulted in a growing number of individuals facing PIU. Estimates suggest the global prevalence of PIU varies between 20.0% and 44.6%, depending on the region [29–30]. Longitudinal studies have shown that PIU increases the risk of anxiety [31, 32, 33]. Zimmermann et al. found through longitudinal tracking data analysis that PIU is significantly associated with an increased risk of anxiety in college students [31]. Gansnerd et al.‘s cohort study, based on data collected through an app-based ecological momentary assessment protocol, revealed that PIU significantly increases and is associated with elevated anxiety risk [32]. Wartberg et al.‘s longitudinal study, analyzing data from 586 middle school students in the Northeast U.S., found that PIU is linked to an increased risk of social anxiety [33]. This growing prevalence has heightened concerns about PIU’s adverse effects on internet users’ psychological well-being. Both bedtime smartphone use and PIU are associated with a higher risk of anxiety, suggesting a potential interaction between them [4, 5, 6, 31, 32, 33]. Strengthening our understanding of how various risk factors interact can help identify variables that can modulate these risks. This knowledge can then be used to develop more precise risk interventions tailored to different population characteristics [34]. Therefore, further investigation is needed to explore whether reducing PIU can alleviate the association between bedtime smartphone use and anxiety.

Based on the above research background, this study proposes the following hypotheses: (1) Bedtime smartphone use increases the risk of anxiety in users. (2) The impact of bedtime smartphone use on anxiety symptoms differs by gender, with the effect being more pronounced in females than in males. (3) PIU interacts with the impact of bedtime smartphone use on anxiety. Based on our research hypotheses, the study conducted the following analyses: (1) Logistic regression and linear regression models were used separately to analyze the relationship between bedtime smartphone use and anxiety risk, as well as the severity of anxiety in the general population. Using network analysis to further explore the underlying mechanisms of the association between bedtime smartphone use and anxiety symptoms. (2) Network comparison testing (NCT) was used to explore gender differences in the association between bedtime smartphone use and anxiety symptoms. (3) Interaction analysis was conducted using logistic regression models to examine the interaction effect of PIU on the association between bedtime smartphone use and anxiety symptoms. It is worth noting that the use of electronic devices at night can adversely affect users’ sleep, thereby increasing their risk of anxiety [12–13]. To mitigate the detrimental effects of bedtime smartphone use on sleep quality, Gradisar et al. recommended avoiding smartphone usage for one hour before bedtime [35]. Therefore, in exploring the association between bedtime smartphone use and anxiety risk and the severity of anxiety, this study referred to Gradisar et al.‘s research and transformed the duration of bedtime smartphone use into a binary variable using a one-hour threshold for analysis [35]. Furthermore, following Demetrovics et al.‘s research, participants were grouped based on a cutoff PIU score of 15, separating individuals into those with and without PIU [36]. This variable handling strategy simplifies the variable structure, enhances the interpretative validity of the interaction analysis, and provides clear classification criteria for developing targeted intervention strategies. By gaining a preliminary understanding of these associations, this study aimed to promote healthy smartphone habits among users and lay the groundwork for future longitudinal research.

## Materials and methods

### Data source and participants

This study utilized data from the Chinese Psychological and Behavioural Study of the Population (PBICR), a nationwide, multicenter, large-sample cross-sectional survey conducted in China [37–39]. PBICR provides a comprehensive and systematic dataset on the mental health and health behaviors of the Chinese population, offering valuable support for research in these areas. The survey was conducted from June 20, 2022, to August 31, 2022, utilizing a stratified and quota sampling method. It encompassed 148 cities, 202 districts/counties, 390 townships/streets, and 780 communities/villages across 23 provinces, 5 autonomous regions, and 4 municipalities directly under China’s Central Government (excluding Hong Kong, Macao, and Taiwan). Trained investigators administered the questionnaire face-to-face to participants. The questionnaire covered eight aspects: demographics, individual health status, family information, social environment, psychological assessment scales, behavioral assessment scales, and attitudes toward societal hot topics. This study was registered with the China Clinical Trial Registry (ChiCTR) (ChiCTR2200061046) [40, 41].

### Definition of variables

#### Bedtime smartphone use

In this study, bedtime smartphone use was recorded as a single item in the database, where participants were asked to report the average number of minutes per day they spent using a smartphone before going to bed over the past week. The measurement options for this item were set in nine gradient intervals, each with a 15-minute range: 0–15 min, 16–30 min, 31–45 min, 46–60 min, 61–75 min, 76–90 min, 91–105 min, 106–120 min, and over 120 min. This interval design, with 15-minute increments, aims to reduce cognitive load for participants when reporting, minimize recall bias, and improve the accuracy of time estimates [42–43]. Participants were required to select the option that best matched their actual usage.

#### Anxiety status

Participants’ anxiety status was assessed using the Generalized Anxiety Disorder 7 (GAD-7) scale recorded in the database. GAD-7 is a self-assessment scale comprising seven items of anxiety symptoms developed by Spitzer et al. [44]. The details of the seven symptom program are as follows: GAD1: feeling nervous, anxious, or on edge, GAD2: inability to stop or control worrying, GAD3: worrying too much about a variety of things, GAD4: difficulty relaxing, GAD5: Inability to sit still due to restlessness, GAD6: becoming annoyed or easily irritated, GAD7: feeling scared because something terrible seems to be about to happen. The scale aims to measure the severity of anxiety symptoms in participants over the past two weeks and their impact on daily functioning. The Likert four-point scoring (0–3) is used to score each symptom item, with a total score ranging from 0 to 21. Different scores represent varying levels of anxiety: 0–4 (no anxiety), 5–9 (mild anxiety), 10–14 (moderate anxiety), and ≥ 15 (severe anxiety). In this study, patients with a score of ≤ 4 were defined as “not anxious” and those with a score of > 4 were defined as“anxious” [45]. The scale has demonstrated good reliability. In previous studies, the GAD-7 showed a Cronbach’s alpha of 0.87 [46], while in the current study, the GAD-7 exhibited a Cronbach’s alpha of 0.84.

#### Problematic internet use (PIU)

PIU was assessed using the PIUQ-SF-6 scale in this study. The PIUQ-SF-6 is a brief scale consisting of six items designed to assess problematic internet use. It evaluates three dimensions: obsession, neglect, and control impairment. Obsession (refers to the obsessive thinking about the internet and the mental symptoms caused by the withdrawal of internet use), neglect (refers to the neglect of basic needs and everyday activities), and control disorder (refers to the difficulties in controlling internet use). Each subscale have two items, and each item was scored with five responses, rated from 1 (never) to 5 (always/almost always). Participants respond using a five-point Likert scale (1–5) for each item. Scores range from 6 to 30, with higher scores indicating a greater risk of PIU [47]. The scale has demonstrated good reliability. In previous studies, the PIUQ-SF-6 showed a Cronbach’s alpha of 0.77 [36], while in the current study, the PIUQ-SF-6 exhibited a Cronbach’s alpha of 0.89.

### Statistical analysis

Descriptive analysis, comparison of general information, logistic regression analysis, linear regression analysis, and interaction analysis were performed using SPSS 26.0. Continuous variables were normally distributed. Descriptive statistics (mean, standard deviation) were used for quantitative data, and frequency and proportion were used for categorical data. Between-group comparisons were made using t-tests and one-way ANOVA. Pearson correlation analysis was used to assess the relationship between two continuous variables. The r value is the Pearson correlation coefficient, used to measure the linear relationship between two continuous variables.

Logistic regression and linear regression models were used separately to analyze the relationship between bedtime smartphone use and anxiety risk, as well as the severity of anxiety in the general population. Additionally, logistic regression was used to examine the interaction effect of PIU on the association between bedtime smartphone use and anxiety symptoms.

Network analysis and network comparison tests (NCT) were conducted using R 4.3.1 software. Network analysis to further explore the underlying mechanisms of the association between bedtime smartphone use and anxiety symptoms. Network comparison testing (NCT) was used to explore gender differences in the association between bedtime smartphone use and anxiety symptoms. Network comparison tests (NCT) were conducted using the NetworkComparisonTest package [21]. The “bootnet” package was used to estimate the network and calculate Pearson correlation coefficients between variables. Least Absolute Shrinkage and Selection Operator (LASSO) regularization and Extended Bayesian Information Criterion (EBIC) were employed to optimize network estimation and reduce noise [26, 48]. The Fruchterman-Reingold algorithm was used for network layout [49]. Within the network, marginal weight values represent the strength of bedtime smartphone use duration’s influence on anxiety symptoms. Strength, a highly stable and replicable centrality index, was calculated using the centrality_plot function to evaluate each node’s overall importance [50]. The network structure’s robustness was evaluated using a bootstrapped case-dropping method. The Correlation Stability (CS) coefficient value represents the maximum proportion of cases that can be removed while maintaining network stability. A CS value greater than 0.25 indicates a stable and reliable network structure [51].

We adopted a two-tailed approach for all statistical tests conducted in this study, with a *p*-value of less than 0.05 considered statistically significant.

## Results

### Baseline characteristics

This study included 30,504 participants from the PBICR 2022 cross-sectional survey. Among these participants, 15,098 (49.50%) reported experiencing anxiety, with a mean anxiety score of 4.99 (SD = 4.77). Among the anxiety symptoms assessed by the GAD-7 scale, the mean scores (standard deviation, SD) were as follows: “Feeling nervous, anxious, or on edge (GAD1)” scored 0.81 (SD = 0.77), “Inability to stop or control worrying (GAD2)” 0.70 (SD = 0.79), “Worrying too much about a variety of things (GAD3)” 0.78 (SD = 0.81), “Difficulty relaxing (GAD4)” 0.74 (SD = 0.81), “Inability to sit still due to restlessness (GAD5)” 0.63 (SD = 0.76), “Becoming annoyed or easily irritated (GAD6)” 0.75 (SD = 0.79), and “Feeling scared because something terrible seems to be about to happen (GAD7)” 0.58 (SD = 0.77) (Table 1).

Males comprised 56.63% (17,275) of the participants. The median age of the participants was 36.08 years (SD = 18.12). Additionally, 27.8% of participants reported using their smartphones for more than one hour before bedtime. Furthermore, 1,081 individuals (35.40%) met the criteria for PIU (Table 2).

**Table 1:** Anxiety status among participants

Variables		Mean/*N*	SD/%
Anxiety	Yes	15,098	49.50
	No	15,406	50.50
Anxiety Symptoms (scores)	Total	4.99	4.77
	GAD1: Feeling nervous, anxious, or on edge	0.81	0.77
	GAD2: Inability to stop or control worrying	0.70	0.79
	GAD3: Worrying too much about a variety of things	0.78	0.81
	GAD4: Difficulty relaxing	0.74	0.81
	GAD5: Inability to sit still due to restlessness	0.63	0.76
	GAD6: Becoming annoyed or easily irritated	0.75	0.79
	GAD7: Feeling scared because something terrible seems to be about to happen	0.58	0.77

**Table 2:** Comparison of anxiety scores among participants with different characteristics

Variables		Mean/*N*	SD/%	t/F/*r*	*P*
Gender (%)	Male	17,275	56.63	-2.712^a^	0.007
	Female	13,229	43.37		
Age (years)		36.08	18.12	-0.103^c^	<0.001
BMI (kg/m^2^)	≥ 24	6567	21.50	-6.817^a^	<0.001
	<24	23,937	78.50		
Residence (%)	Cities and towns	8444	27.68	-3.683^a^	<0.001
	Rural	22,060	72.32		
Marriage Status (%)	Married	16,355	53.62	-19.485^a^	<0.001
	Others	14,149	46.38		
Monthly per capita family income (yuan)	≥ 4000	15,263	50.04	0.035^a^	0.972
	< 4000	15,241	49.96		
Family Type (%)	Nuclear family	15,885	52.08	34.432^b^	<0.001
	Couple family	6163	20.20		
	Others	8456	27.72		
Suffering from chronic illness (%)	Yes	23,693	77.67	13.966^a^	<0.001
	No	6811	22.33		
Drinking (%)	Yes	21,312	69.87	11.838^a^	<0.001
	No	9192	30.13		
Smoking (%)	Yes	25,693	84.23	7.210^a^	<0.001
	No	4811	15.77		
Drinking tea (%)	Yes	14,899	48.84	9.901^a^	<0.001
	No	15,605	51.16		
Drinking coffee (%)	Yes	22,629	74.18	18.004^a^	<0.001
	No	7875	25.82		
Duration of bedtime smartphone use (hours)	> 1	8490	27.80	24.048^a^	<0.001
	≤ 1	22,014	72.20		
Length of sleep (hours)	> 6	22,480	73.70	29.046^a^	<0.001
	≤ 6	8024	26.30		
Sleep quality (%)	Good	5261	17.25	-41.407^a^	<0.001
	Poor	25,243	82.75		
PIU Total Scores		12.31	5.61	0.454^c^	<0.001

Abbreviations: BMI, Body Mass Index; PIU, problematic internet use

^a^ for t, ^b^ for F, ^c^ for r

### The relationship between the bedtime smartphone use and risk of anxiety

After adjusting for covariates in all three models, the logistic regression results revealed that, compared to participants who used their smartphones for one hour or less before bedtime, using smartphones for more than one hour before bedtime was associated with a 9.1% higher likelihood of experiencing anxiety (OR = 1.091) (Table 3).

**Table 3:** Logistic regression analyses of the association between duration of smartphone use before bedtime and the risk of anxiety

Model Adjustment	β	SE	Wald χ^2^value	*P*-value	OR(95%CI)
Model 1	0.569	0.026	481.987	<0.001	1.767(1.680,1.859)
Model 2	0.490	0.027	323.236	<0.001	1.633(1.548,1.722)
Model 3	0.087	0.031	7.978	0.005	1.091(1.027,1.158)

Model 1 was unadjusted; Model 2 was adjusted for socio-demographic factors, including gender, age, BMI, residence, marital status, monthly per capita household income, and family type; Model 3 was adjusted for diseases factor, behavioral factors and socio-demographic factors, including all variables in Model 2 as well as whether suffering from chronic illness, drinking status, smoking status, drinking tea or not, Drinking coffee or not, length of sleep, sleep quality, and PIU status

### Relationship between the duration of bedtime smartphone use and the severity of anxiety

A multiple linear regression model was used to analyze the association between the duration of bedtime smartphone use and the severity of anxiety (eTable 1 in Supplement). Even after controlling for covariates, the duration of bedtime smartphone use was positively correlated with anxiety severity (β = 0.116, *P* < 0.001).

### Network analysis of the duration of bedtime smartphone use and anxiety symptoms

**Fig. 1:**
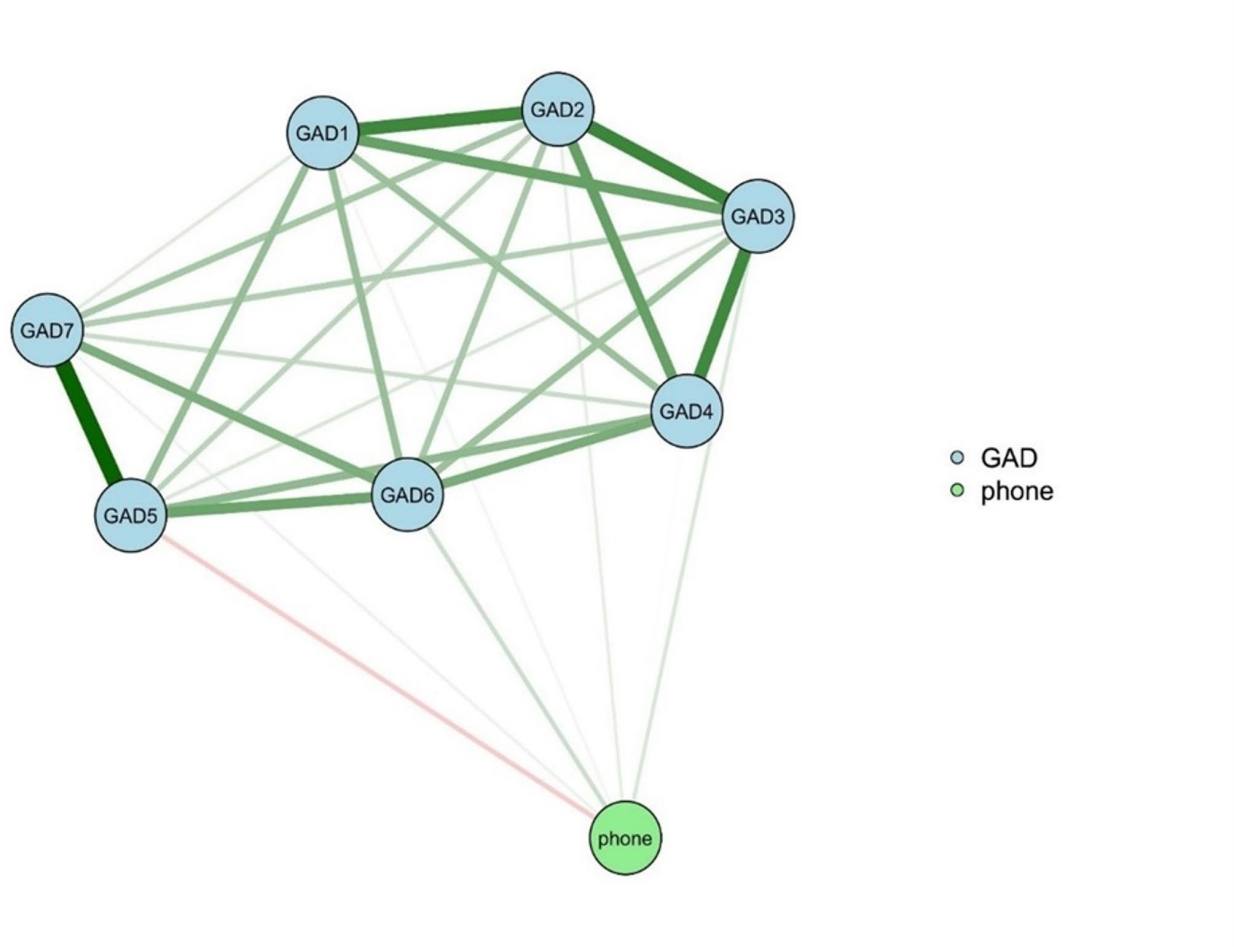
Network Of The Duration Of Bedtime Smartphone Use And Anxiety Symptoms. GAD1: feeling nervous, anxious, or on edge. GAD2: inability to stop or control worrying. GAD3: worrying too much about a variety of things. GAD4: difficulty relaxing. GAD5: inability to sit still due to restlessness. GAD6: becoming annoyed or easily irritated. GAD7: feeling scared because something terrible seems to be about to happen. phone: the duration of bedtime smartphone us

The duration of bedtime smartphone use showed a negative correlation with GAD-5 (inability to sit still due to restlessness) and a positive correlation with the remaining six anxiety symptoms (Fig. 1). Details on network accuracy and stability are presented in the supplemental eResults.

Among all anxiety symptoms in the network, GAD2 (inability to stop or control worrying) exhibited the highest strength, followed by GAD3 (worrying too much about various things) (eFigure 1). Interestingly, the strongest marginal weight value was observed between bedtime smartphone use duration and GAD6 (becoming easily annoyed/irritated) (eTable 2 in Supplement). Detailed results regarding network accuracy and stability are presented in the supplemental materials (eResults, eFigures 2–4).

### Network analysis of the duration of bedtime smartphone use and anxiety symptoms in different genders

**Fig. 2:**
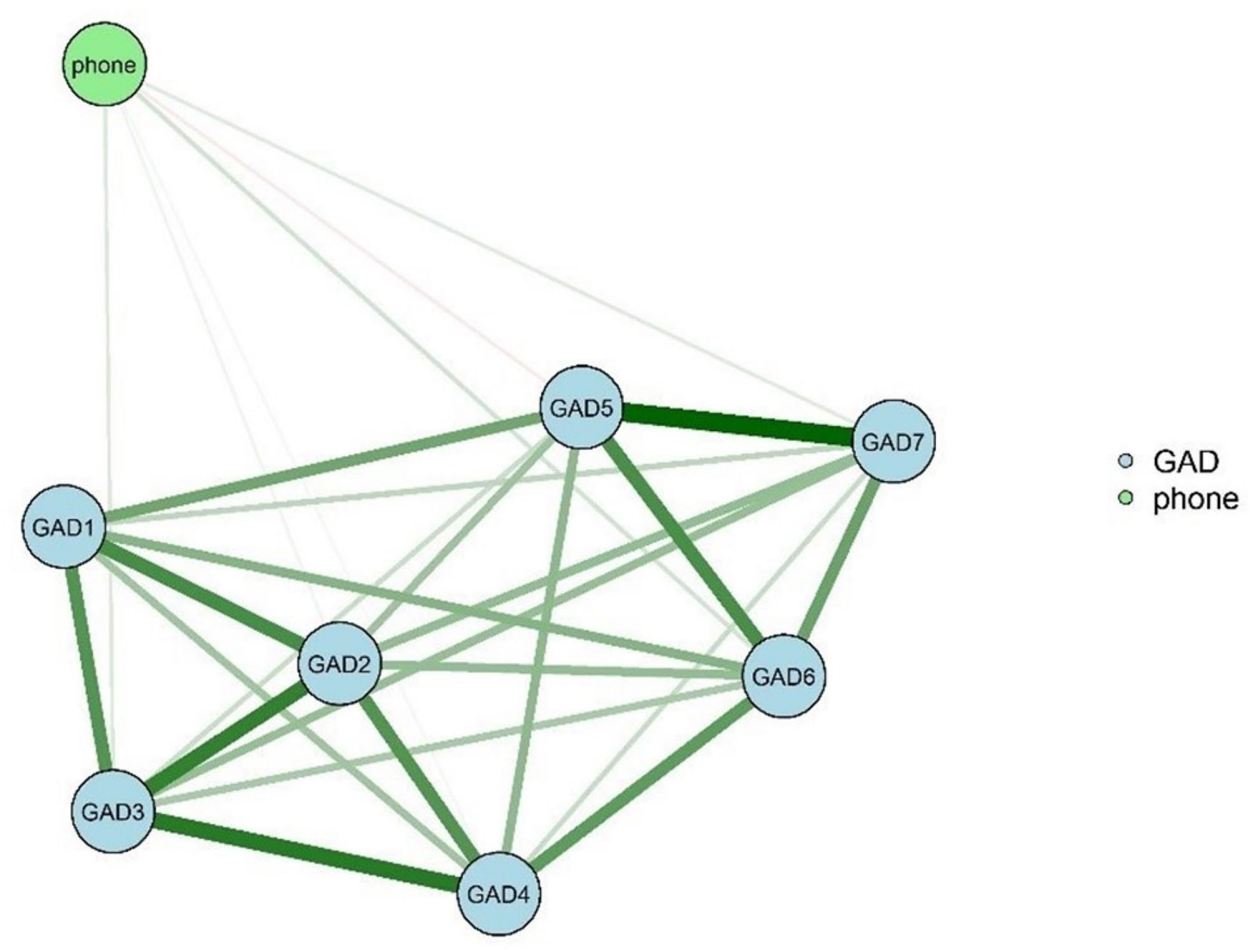
Network Of The Duration Of Bedtime Smartphone Use And Anxiety Symptoms In Males. GAD1: feeling nervous, anxious, or on edge. GAD2: inability to stop or control worrying. GAD3: worrying too much about a variety of things. GAD4: difficulty relaxing. GAD5: inability to sit still due to restlessness. GAD6: becoming annoyed or easily irritated. GAD7: feeling scared because something terrible seems to be about to happen. phone: the duration of bedtime smartphone us

**Fig. 3:**
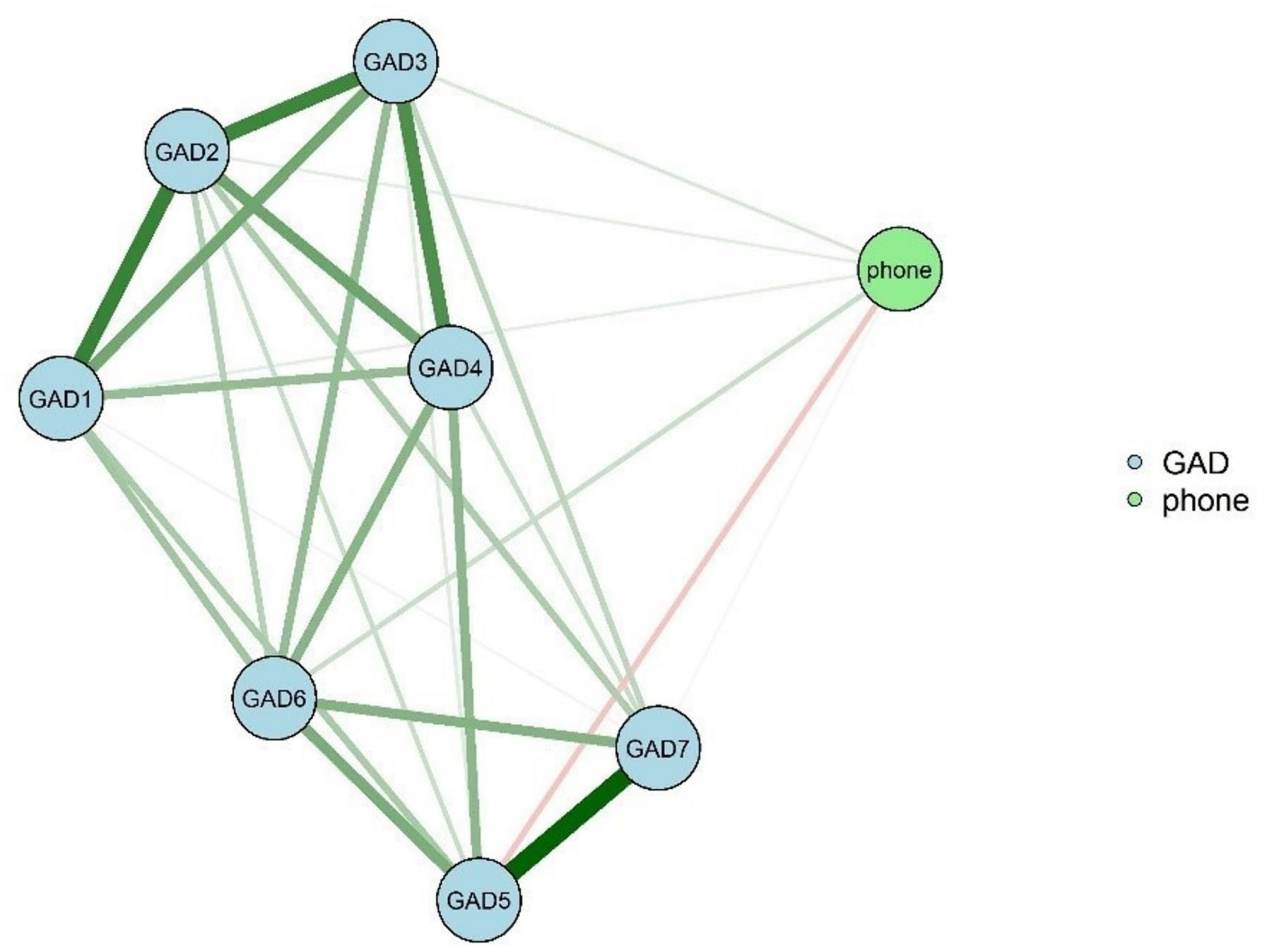
Network Of The Duration Of Bedtime Smartphone Use And Anxiety Symptoms In Females. GAD1: feeling nervous, anxious, or on edge. GAD2: inability to stop or control worrying. GAD3: worrying too much about a variety of things. GAD4: difficulty relaxing. GAD5: inability to sit still due to restlessness. GAD6: becoming annoyed or easily irritated. GAD7: feeling scared because something terrible seems to be about to happen. phone: the duration of bedtime smartphone us

Figures 2 and 3 depict the separate networks for males and females, respectively, revealing the associations between bedtime smartphone use duration and anxiety symptoms. Notably, the female network exhibited a higher strength for GAD4 (inability to relax) compared to the male network (males = 0.948, females = 0.986, *P* = 0.007) (eFigure 6 in Supplement). Conversely, the male network showed a higher strength for GAD7 (feeling scared of something terrible happening) compared to the female network (males = 0.948, females = 0.986, *P* = 0.007) (eFigure 7 in Supplement). Detailed results regarding network comparison, accuracy, and stability for different genders are presented in the supplemental materials (eResults, eFigs. 5, 6, 7, 8, 9, 10, 11, 12 and 13).

The analysis revealed significant gender differences in the association between bedtime smartphone use duration and anxiety symptoms across three connections within the networks (phone-GAD1, phone-GAD5, phone-GAD6; *P* = 0.003, 0.001, and 0.04, respectively). For these connections, female participants exhibited higher marginal weight values compared to males (eTable 3 and eTable 4 in Supplement).

### The interaction between PIU and bedtime smartphone use

Individuals who reported both bedtime smartphone use of more than 1 h and PIU were associated with a 276.2% higher likelihood of experiencing anxiety. Those who reported bedtime smartphone use of more than 1 h and did not have PIU were associated with a 35.3% lower likelihood of experiencing anxiety (OR = 0.647) (Table 4).

**Table 4:** The interaction between PIU and bedtime smartphone use

	Model Adjustment	$\:\varvec{\beta\:}$	SE	Wald χ^2^value	*P*-value	OR(95%CI)
bedtime smartphone use duration>1 h* with PIU	Model 1	1.222	0.036	1165.088	<0.001	3.394(3.164,3.641)
	Model 2	1.163	0.037	994.896	<0.001	3.200(2.977,3.440)
	Model 3	1.016	0.038	714.658	<0.001	2.762(2.564,2.976)
bedtime smartphone use duration>1 h* without PIU	Model 1	-0.289	0.035	69.997	<0.001	0.749(0.700,0.802)
	Model 2	-0.367	0.035	109.124	<0.001	0.693(0.647,0.742)
	Model 3	-0.436	0.037	142.601	<0.001	0.647(0.602,0.695)

Model 1 was unadjusted; Model 2 was adjusted for socio-demographic factors, including gender, age, BMI, residence, marital status, monthly per capita household income, and family type; Model 3 was adjusted for clinical factors, behavioral factors and socio-demographic factors, including all variables in Model 2 as well as whether suffering from chronic illness, drinking status, smoking status, drinking tea or not, drinking coffee or not, length of sleep, sleep quality

## Discussion

This study found using smartphones before bedtime was associated with a higher likelihood of experiencing anxiety and was positively correlated with the severity of anxiety. Within the network analysis, “inability to stop worrying (GAD2)” and “worrying too much (GAD3)” showed the strongest associations with bedtime smartphone use. Interestingly, bedtime smartphone use negatively correlated with “inability to sit still (GAD5)” but positively correlated with other anxiety symptoms, especially “becoming easily annoyed (GAD6)”, which had the strongest edge weight. Gender differences emerged in the network: women showed stronger associations with “difficulty relaxing (GAD4)”, while men exhibited higher intensity for “fear of something terrible happening (GAD7)”. Additionally, women had stronger edge weights between bedtime smartphone use and “nervousness (GAD1)”, “restlessness (GAD5)”, and “irritability (GAD6)” than men. The study also found that PIU moderated the relationship between bedtime smartphone use and anxiety. Individuals who used smartphones for more than 1 h before bed and had PIU were more likely to experience anxiety, those who used smartphones for more than 1 h and did not have PIU were less likely to experience anxiety.

Bedtime smartphone use has become a prevalent lifestyle habit [3]. Prior research suggests a link between bedtime smartphone use and a higher risk of anxiety disorders [7]. Similar to our findings, previous studies have shown a significant correlation between overall screen time and the severity of anxiety symptoms [52]. Several explanations exist for the detrimental effects of bedtime smartphone use on anxiety. Firstly, Screen time may replace valuable time spent with others, harming social connections and emotional well-being [53]. Secondly, using devices at night exposes users to blue light, which delays melatonin release, increases arousal, disrupts sleep, and raises anxiety levels [12–13]. Thirdly, the content viewed before bed also plays a role; for example, exposure to cyberbullying online can increase anxiety. Moreover, seeing idealized content may trigger upward social comparisons, further contributing to anxiety and negatively affecting mental and social health [54–55].

This study found that “inability to stop or control worrying” (GAD2) and “worrying too much about a variety of things” (GAD3) exhibited the strongest intensity among anxiety symptoms, indicating that worry may be the most significant and prevalent symptom among bedtime smartphone users. Research indicates that using social media before bed is associated with anxiety. Nearly 50% of smartphone users spend more than an hour on social networks before sleeping [56]. Prolonged use can cause social media fatigue, which is closely linked to anxiety and results in various worrying emotions [57]. Interestingly, the network analysis showed a negative correlation between bedtime smartphone use and “inability to sit still due to restlessness” (GAD5). Lepp et al. found that high-frequency smartphone users are generally more sedentary. Since GAD5 reflects anxiety related to movement, it may not fully capture the link between bedtime smartphone use and anxiety [58]. Additionally, bedtime smartphone use can significantly disrupt sleep patterns. Using social media and apps before bed can delay sleep, disrupt melatonin secretion, interfere with the sleep-wake cycle, shorten sleep duration, and increase irritability [59, 60, 61]. This explains why our study found that “becoming easily annoyed or irritated (GAD6)” had the strongest link with bedtime smartphone use.

This study revealed gender differences in the network structure of bedtime smartphone use and anxiety symptoms. In females, “difficulty relaxing” (GAD4) showed higher centrality in the network, and the connections between bedtime smartphone use and symptoms like “feeling nervous” (GAD1), “inability to sit still” (GAD5), and “becoming easily annoyed” (GAD6) were stronger than in males. These differences may be due to females being more sensitive to emotional impacts, more frequent social media use, and having fewer coping mechanisms for stress [20, 21, 22, 23]. Additionally, females are more affected by the negative impacts of bedtime smartphone use on sleep quality [24–25]. In contrast, males showed a higher intensity for “feeling scared because something terrible seems to be about to happen” (GAD7). Research suggests this could be due to males experiencing a higher fear of missing out (FOMO), which drives a desire to stay connected on social media and fear missing enjoyable experiences [62–63].

Research shows a positive correlation between PIU and anxiety, with PIU individuals scoring higher on anxiety measures than non-PIU individuals [31, 32, 33]. The compensatory internet use theory suggests that socially anxious people may increase their anxiety by using the internet as a substitute for real-life social interactions [64, 65, 66]. Our study found that PIU exacerbates the negative effects of bedtime smartphone use on anxiety. Specifically, individuals with PIU experience a greater increase in anxiety from smartphone use before bed compared to those without PIU. This indicates that reducing PIU could help mitigate the adverse effects of prolonged bedtime smartphone use.

This study has several limitations. First, all participants were Chinese, so the findings may not be generalizable to other populations. Second, the study used self-reported data, which may be subject to misclassification, recall issues, and response biases. Third, as a cross-sectional observational study, it cannot establish causality. While we accounted for many confounding factors, reverse causation and unmeasured confounders may still have influenced the results.

Our research highlights the need to reduce bedtime smartphone use. Individuals can start by being mindful of their nightly phone habits and cutting down on screen time before sleep. On a societal level, public health initiatives can promote healthy digital habits in schools and workplaces. Technology companies can also help by creating features that encourage responsible phone use, such as bedtime reminders or restricted modes. To spread these findings, engaging content on platforms like WeChat and articles in mainstream media can raise awareness and promote positive behavioral change.

## Conclusions

This study demonstrates that bedtime was associated with a higher likelihood of developing anxiety and was positively correlated with the severity of anxiety in the general population. Worry-related symptoms showed the strongest association with bedtime smartphone use. Furthermore, the association between bedtime smartphone use and anxiety symptoms was stronger in women than in men. Additionally, our findings revealed that PIU moderated the relationship between bedtime smartphone use and anxiety. Using smartphones for more than 1 h before bedtime, combined with PIU, was associated with a higher likelihood of developing anxiety, while using a smartphone for more than 1 h before bedtime, without PIU, was associated with a lower likelihood of developing anxiety. This finding provides a potential basis for developing interventions that alleviate anxiety symptoms in the general population by targeting PIU reduction. It also provides valuable direction and a foundation for future longitudinal studies to further explore these relationships.

## Electronic Supplementary Material

Below is the link to the electronic supplementary material.

Supplementary Material 1

## Data Availability

The datasets used and/or analysed during the current study available from the corresponding author on reasonable request.

## Abbreviations

PBICR: Chinese Psychological and Behavioural Study of the Population
BMI: Body mass index

## References

[1] Statista. Global smartphone penetration rate as share of population from 2016 to 2024. https://www.statista.com/statistics/203734/global-smartphone-penetration-per-capita-since-2005/. Accessed 7 Apr 2025.

[2] Statista. Apr. Number of smartphone users in China from 2018 to 2022 with a forecast until 2027.https://www.statista.com/statistics/467160/forecast-of-smartphone-users-in-china/. Accessed 7 2025.

[3] Statista. Apr. People’s usual activities within the last hour before sleep in China as of 2020. https://www.statista.com/statistics/1291839/china-common-activities-before-sleep/. Accessed 7 2025.

[4] Xiong W. A study on anxiety symptoms and their influencing factors among middle school students in Ganzhou City. Jilin University; 2020. 10.27162/d.cnki.gjlin.2020.004124.

[5] Feng J. A study on residents’ mental health and its related factors based on the theory of health ecology. Huazhong University of Science and Technology; 2024. 10.27157/d.cnki.ghzku.2024.000830.

[6] Wang J. A comparison of sleep characteristics and emotional symptoms among college students with different sleep chronotypes: A cross-sectional study. South Med Univ. 2023. 10.27003/d.cnki.gojyu.2023.001097.

[7] Mei X, Hu Z, Zhou D, et al. Sleep patterns, mobile phone use and psychological symptoms among adolescents in coastal developed City of China: an exploratory cross-sectional study. Sleep Biol Rhythms. 2019;17(2):233–41. 10.1007/s41105-019-00208-1.

[8] Maftei A, Măirean C. Put your phone down! Perceived phubbing, life satisfaction, and psychological distress: the mediating role of loneliness. BMC Psychol. 2023;11(1):332. 10.1186/s40359-023-01359-0. 37828557 10.1186/s40359-023-01359-0 PMC10571372

[9] Bobo WV, Grossardt BR, Virani S, et al. Association of depression and anxiety with the accumulation of chronic conditions. JAMA Netw Open. 2022;5(5):e229817. 10.1001/jamanetworkopen.2022.9817. 35499825 10.1001/jamanetworkopen.2022.9817 PMC9062691

[10] Fan M, Huang Y, Qalati SA, et al. Effects of information overload, communication overload, and inequality on digital distrust: A Cyber-Violence behavior mechanism. Front Psychol. 2021;12:643981. 10.3389/fpsyg.2021.643981. 33959073 10.3389/fpsyg.2021.643981 PMC8093436

[11] Loh XK, Lee VH, Loh XM, et al. The dark side of mobile learning via social media: how bad can it get?? Inf Syst Front. 2022;24(6):1887–904. 10.1007/s10796-021-10202-z. 34658660 10.1007/s10796-021-10202-z PMC8501931

[12] Hamilton JL, Lee W. Associations between social media, bedtime technology use rules, and daytime sleepiness among adolescents: Cross-sectional findings from a nationally representative sample. JMIR Ment Health. 2021;8(9):e26273. 10.2196/26273. 34524967 10.2196/26273 PMC8482309

[13] Ghrouz AK, Noohu MM, Dilshad MM, et al. Physical activity and sleep quality in relation to mental health among college students. Sleep Breath. 2019;23(2):627–34. 10.1007/s11325-019-01780-z. 30685851 10.1007/s11325-019-01780-z

[14] Acuña A, Morales S, Uriarte-Gaspari L, et al. Increased default mode network activation in depression and social anxiety during upward social comparison. Soc Cogn Affect Neurosci. 2025;20(1):nsaf012. 10.1093/scan/nsaf012. 39882939 10.1093/scan/nsaf012 PMC11792650

[15] Hou J, Zhu Y, Fang X. Mobile phone addiction and depression: the multiple mediating effects of social anxiety and negative emotional information attention bias. Acta Physiol Sinica. 2021;53(04):362–73.

[16] Weinstein A, Abu HB, Timor A, et al. Delay discounting, risk-taking, and rejection sensitivity among individuals with internet and video gaming disorders. J Behav Addict. 2016;5(4):674–82. 10.1556/2006.5.2016.081. 27958761 10.1556/2006.5.2016.081 PMC5370373

[17] Zhang G, Yang X, Tu X, et al. Prospective relationships between mobile phone dependence and mental health status among Chinese undergraduate students with college adjustment as a mediator. J Affect Disord. 2020;260:498–505. 10.1016/j.jad.2019.09.047. 31539686 10.1016/j.jad.2019.09.047

[18] Thomée S, Eklöf M, Gustafsson E, et al. Prevalence of perceived stress, symptoms of depression and sleep disturbances in relation to information and communication technology (ICT) use among young adults–an explorative prospective study. Comput Hum Behav. 2007;23(3):1300–21. 10.1016/j.chb.2004.12.007.

[19] Thomée S, Härenstam A, Hagberg M. Mobile phone use and stress, sleep disturbances, and symptoms of depression among young adults - a prospective cohort study. BMC Public Health. 2011;11(1):66. 10.1186/1471-2458-11-66. 21281471 10.1186/1471-2458-11-66 PMC3042390

[20] Li SH, Graham BM. Why are women so vulnerable to anxiety, trauma-related and stress-related disorders? The potential role of sex hormones. Lancet Psychiatry. 2017;4(1):73–82. 10.1016/S2215-0366(16)30358-3. 27856395 10.1016/S2215-0366(16)30358-3

[21] Wang J, Luo Y, Yan N, et al. Network structure of mobile phone addiction and anxiety symptoms among rural Chinese adolescents. BMC Psychiatry. 2023;23(1):491. 10.1186/s12888-023-04971-x. 37430241 10.1186/s12888-023-04971-x PMC10332091

[22] Hankin BL, Abramson LY. Development of gender differences in depression: description and possible explanations. Ann Med. 1999;31(6):372–9. 10.3109/07853899908998794. 10680851 10.3109/07853899908998794

[23] Li P, Zhuo Q. Emotional straying: flux and management of women’s emotions in social media. PLoS ONE. 2023;18(12):e0295835. 10.1371/journal.pone.0295835. 38091307 10.1371/journal.pone.0295835 PMC10718421

[24] Amez S, Vujić S, Soffers P, et al. Yawning while scrolling? Examining gender differences in the association between smartphone use and sleep quality. J Sleep Res. 2020;29(6):e12971. 10.1111/jsr.12971. 31919946 10.1111/jsr.12971

[25] Spilková J, Chomynová P, Csémy L. Predictors of excessive use of social media and excessive online gaming in Czech teenagers. J Behav Addict. 2017;6(4):611–9. 10.1556/2006.6.2017.064. 29039223 10.1556/2006.6.2017.064 PMC6034940

[26] Zhang P, Wang L, Zhou Q, et al. A network analysis of anxiety and depression symptoms in Chinese disabled elderly. J Affect Disord. 2023;333:535–42. 10.1016/j.jad.2023.04.065. 37086797 10.1016/j.jad.2023.04.065

[27] Phua DY, Chen H, Chong YS, et al. Network analyses of maternal Pre- and Post-Partum symptoms of depression and anxiety. Front Psychiatry. 2020;11:785. 10.3389/fpsyt.2020.00785. 32848949 10.3389/fpsyt.2020.00785 PMC7424069

[28] Reed P, Davies A, Evans K, et al. Longitudinal relationship between problematic internet use with loneliness during and after COVID-19 social restrictions: short title: internet use and loneliness. Psychiatry Res. 2023;323:115148. 10.1016/j.psychres.2023.115148. 36905904 10.1016/j.psychres.2023.115148

[29] Chia DXY, Ng CWL, Kandasami G, et al. Prevalence of internet addiction and gaming disorders in Southeast Asia: A Meta-Analysis. Int J Environ Res Public Health. 2020;17(7):2582. 10.3390/ijerph17072582. 32283803 10.3390/ijerph17072582 PMC7177828

[30] Endomba FT, Demina A, Meille V, et al. Prevalence of internet addiction in Africa: A systematic review and meta-analysis. J Behav Addict. 2022;11(3):739–53. 10.1556/2006.2022.00052. 35984734 10.1556/2006.2022.00052 PMC9872524

[31] Zimmermann M, Bledsoe C, Papa A. Initial impact of the COVID-19 pandemic on college student mental health: A longitudinal examination of risk and protective factors. Psychiatry Res. 2021;305:114254. 10.1016/j.psychres.2021.114254. 34763271 10.1016/j.psychres.2021.114254 PMC8556872

[32] Gansner M, Nisenson M, Lin V, et al. Problematic internet use before and during the COVID-19 pandemic in youth in outpatient mental health treatment: App-Based ecological momentary assessment study. JMIR Ment Health. 2022;9(1):e33114. 10.2196/33114. 35089157 10.2196/33114 PMC8797151

[33] Wartberg L, Lindenberg K. Predictors of spontaneous remission of problematic internet use in adolescence: A One-Year Follow-Up study. Int J Environ Res Public Health. 2020;17(2):448. 10.3390/ijerph17020448. 31936677 10.3390/ijerph17020448 PMC7014287

[34] Zhao M, Qu T, Li Y, et al. Interaction effects of anxiety and outdoor activity spaces on frailty among nursing home residents in Jinan, China: is there a gender difference? Front Public Health. 2023;11:1133340. 10.3389/fpubh.2023.1133340. 36908457 10.3389/fpubh.2023.1133340 PMC9999001

[35] Gradisar M, Wolfson AR, Harvey AG, et al. The sleep and technology use of Americans: findings from the National sleep foundation’s 2011 sleep in America poll. J Clin Sleep Med. 2013;9(12):1291–9. 10.5664/jcsm.3272. 24340291 10.5664/jcsm.3272 PMC3836340

[36] Demetrovics Z, Király O, Koronczai B, et al. Psychometric properties of the problematic internet use questionnaire Short-Form (PIUQ-SF-6) in a nationally representative sample of adolescents. PLoS ONE. 2016;11(8):e0159409. 10.1371/journal.pone.0159409. 27504915 10.1371/journal.pone.0159409 PMC4978438

[37] Wang Y, Kaierdebieke A, Fan S, et al. Study protocol: a cross-sectional study on psychology and behavior investigation of Chinese residents, PBICR. Psychosom Med Res. 2022;4(3):19. 10.53388/202219.

[38] Zhang Y, Fan S, Hui H, et al. Privacy protection for open sharing of psychiatric and behavioral research data: ethical considerations and recommendations. Alpha Psychiatr. 2025;26(1):38759. 10.31083/AP38759. 10.31083/AP38759 PMC11915712 40110382

[39] Liu D, Fan S, Huang X, et al. Study protocol: a national cross-sectional study on psychology and behavior investigation of Chinese residents in 2023. Health Care Sci. 2024;3(6):475–92. 10.1002/hcs2.125. 10.1002/hcs2.125 PMC11671216 39735279

[40] Wu Y, Fan S, Liu D, et al. Psychological and behavior investigation of Chinese residents: concepts, practices, and prospects. Chin Gen Pract J. 2024;1(3):149–56. 10.1016/j.cgpj.2024.07.006.

[41] Yang Y, Fan S, Chen W, et al. Broader open data needed in psychiatry: practice from the psychology and behavior investigation of Chinese residents. Alpha Psychiatr. 2024;25(4):564–5. 10.5152/alphapsychiatry.2024.241804. 10.5152/alphapsychiatry.2024.241804 PMC11443289 39360297

[42] Dillman DA, Smyth JD, Christian LM, Internet. phone, mail, and mixed-mode surveys: the tailored design method. Indiana: Indianapolis; 2014.

[43] Schwarz N. Self-reports: how the questions shape the answers. Am Psychol. 1999;54(2):93. 10.1037/0003-066X.54.2.93.

[44] Spitzer RL, Kroenke K, Williams JBW, et al. A brief measure for assessing generalized anxiety disorder: the GAD-7. Arch Intern Med. 2006;166(10):1092–7. 10.1001/archinte.166.10.1092. 16717171 10.1001/archinte.166.10.1092

[45] Tian Z, Li Y, Zhang N, et al. Dose-response relationship between sedentary time and anxiety and the moderating effect of a 10-min walk: a cross-sectional study. BMC Psychiatry. 2025;25(1):51. 10.1186/s12888-025-06496-x. 39827107 10.1186/s12888-025-06496-x PMC11742805

[46] Guo Yuhang S, Haoran W, Zhuo, et al. Heart rate variability and its influencing factors in an elderly population in Changchun City. Chin J Geriatr. 2024;44(23):5867–71.

[47] Yu Y, Wu Y, Chen P, et al. Associations between personality and problematic internet use among Chinese adolescents and young adults: A network analysis. J Affect Disord. 2024;365:501–8. 10.1016/j.jad.2024.08.069. 39178960 10.1016/j.jad.2024.08.069

[48] Foygel R, Drton M. Extended Bayesian Information Criteria for Gaussian Graphical Models. In: Advances in Neural Information Processing Systems. Vol 23. Curran Associates, Inc.; 2010. Accessed November 23, 2023. https://proceedings.neurips.cc/paper/2010/hash/072b030ba126b2f4b2374f342be9ed44-Abstract.html

[49] Malhotra K, Kempegowda P. Appraising unmet needs and misinformation spread about polycystic ovary syndrome in 85,872 YouTube comments over 12 years: big data infodemiology study. J Med Internet Res. 2023;25:e49220. 10.2196/49220. 37695666 10.2196/49220 PMC10520765

[50] Monteleone AM, Cascino G, Salerno L, et al. The interplay between emotion regulation, interpersonal problems and eating symptoms in individuals with obesity: A network analysis study. J Affect Disord. 2023;324:61–7. 10.1016/j.jad.2022.12.056. 36565965 10.1016/j.jad.2022.12.056

[51] Guo Z, Yang T, Qiu R, et al. Network analysis of the relationships between problematic smartphone use and anxiety, and depression in a sample of Chinese college students. Front Psychiatry. 2023;14:1097301. 10.3389/fpsyt.2023.1097301. 37139318 10.3389/fpsyt.2023.1097301 PMC10149733

[52] Tang S, Werner-Seidler A, Torok M, et al. The relationship between screen time and mental health in young people: A systematic review of longitudinal studies. Clin Psychol Rev. 2021;86:102021. 10.1016/j.cpr.2021.102021. 33798997 10.1016/j.cpr.2021.102021

[53] Richards R, McGee R, Williams SM, et al. Adolescent screen time and attachment to parents and peers. Arch Pediatr Adolesc Med. 2010;164(3):258–62. 10.1001/archpediatrics.2009.280. 20194259 10.1001/archpediatrics.2009.280

[54] Hamm MP, Newton AS, Chisholm A, et al. Prevalence and effect of cyberbullying on children and young people: A scoping review of social media studies. JAMA Pediatr. 2015;169(8):770–7. 10.1001/jamapediatrics.2015.0944. 26098362 10.1001/jamapediatrics.2015.0944

[55] Tiggemann M, Hayden S, Brown Z, et al. The effect of Instagram likes on women’s social comparison and body dissatisfaction. Body Image. 2018;26:90–7. 10.1016/j.bodyim.2018.07.002. 30036748 10.1016/j.bodyim.2018.07.002

[56] Levenson JC, Shensa A, Sidani JE, et al. Social media use before bed and sleep disturbance among young adults in the united States: A nationally representative study. Sleep. 2017;40(9):zsx113. 10.1093/sleep/zsx113. 28934521 10.1093/sleep/zsx113 PMC8205627

[57] IEEE. Towards understanding SNS fatigue: exploration of social experience in the Virtual World. In, Convergence Technology (ICCCT). 2012 7th International Conference on Computing and; 2012. Accessed November 23, 2023. https://ieeexplore.ieee.org/abstract/document/6530327

[58] Lepp A, Barkley JE, Sanders GJ, et al. The relationship between cell phone use, physical and sedentary activity, and cardiorespiratory fitness in a sample of U.S. College students. Int J Behav Nutr Phys Act. 2013;10:79. 10.1186/1479-5868-10-79. 23800133 10.1186/1479-5868-10-79 PMC3693866

[59] Royant-Parola S, Londe V, Tréhout S, et al. The use of social media modifies teenagers’ sleep-related behavior. L’encephale. 2017;44(4):321–doi328. 10.1016/j.encep.2017.03.009. 28602529 10.1016/j.encep.2017.03.009

[60] Van Veen MM, Lancel M, Şener O, et al. Observational and experimental studies on sleep duration and aggression: A systematic review and meta-analysis. Sleep Med Rev. 2022;64:101661. 10.1016/j.smrv.2022.101661. 36064210 10.1016/j.smrv.2022.101661

[61] Song Y, Sznajder K, Cui C, et al. Anxiety and its relationship with sleep disturbance and problematic smartphone use among Chinese medical students during COVID-19 home confinement - A structural equation model analysis. J Affect Disord. 2022;296:315–21. 10.1016/j.jad.2021.09.095. 34600968 10.1016/j.jad.2021.09.095 PMC8484230

[62] Przybylski AK, Murayama K, DeHaan CR, et al. Motivational, emotional, and behavioral correlates of fear of missing out. Comput Hum Behav. 2013;29(4):1841–8. 10.1016/j.chb.2013.02.014.

[63] Elhai JD, Yang H, Montag C. Fear of missing out (FOMO): overview, theoretical underpinnings, and literature review on relations with severity of negative affectivity and problematic technology use. Braz J Psychiatry. 2021;43(2):203–9. 10.1590/1516-4446-2020-0870. 32401865 10.1590/1516-4446-2020-0870 PMC8023172

[64] Kitazawa M, Yoshimura M, Murata M, et al. Associations between problematic internet use and psychiatric symptoms among university students in Japan. Psychiatry Clin Neurosci. 2018;72(7):531–9. 10.1111/pcn.12662. 29652105 10.1111/pcn.12662

[65] Kardefelt-Winther D. A conceptual and methodological critique of internet addiction research: towards a model of compensatory internet use. Comput Hum Behav. 2014;31:351–4. 10.1016/j.chb.2013.10.059.

[66] Ding H, Cao B, Sun Q. The association between problematic internet use and social anxiety within adolescents and young adults: a systematic review and meta-analysis. Front Public Health. 2023;2911:1275723. 10.3389/fpubh.2023.1275723. 10.3389/fpubh.2023.1275723 PMC10570444 37841708

