# Supplementary material for: The association between bedtime smartphone use and anxiety symptoms: a network analysis of Chinese residents
Source: BMC Psychiatry. 2025 May 26;25:545. doi: 10.1186/s12888-025-06961-7 (PMC12107847; doi:10.1186/s12888-025-06961-7)
Supplement: Supplementary file 1 — Supplementary Material 1 [file 12888_2025_6961_MOESM1_ESM.docx]

**Supplementary Content**

**eMethods**

**eResults**

**eTable 1.** Linear Multiple Regression Analysis Of The Association Between Duration Of Smartphone Use Before Bedtime And The Severity Of Anxiety

**eTable 2.** Weighted Adjacency Matrix Of The Duration Of Smartphone Use Before Bedtime And Anxiety Symptoms

**eTable 3.** Weighted Adjacency Matrix Of The Duration Of Smartphone Use Before Bedtime And Anxiety Symptoms In Males

**eTable 4.** Weighted Adjacency Matrix Of The Duration Of Smartphone Use Before Bedtime And Anxiety Symptoms In Females

**eFigure 1.** Node Strength Centrality In The Network

**eFigure 2.** Estimating The Stability Of The Network Structure Using The Case-Drop Subset Bootstrap Method

**eFigure 3.** Difference-In-Difference Test For Node Strength

**eFigure 4.** Bootstrap Difference Test Of Network Edge Weights

**eFigure 5.** Comparison Of Network Attributes Of Participants In Different Gender

**eFigure 6.** Node Strength Centrality In The Network In Males

**eFigure 7.** Node Strength Centrality In The Network In Females

**eFigure 8.** Estimating The Stability Of The Network Structure Using The Case-Drop Subset Bootstrap Method In Males

**eFigure 9.** Estimating The Stability Of The Network Structure Using The Case-Drop Subset Bootstrap Method In Females

**eFigure 10.** Difference-In-Difference Test For Node Strength In Males

**eFigure 11.** Difference-In-Difference Test For Node Strength In Females

**eFigure 12.** Bootstrap Difference Test Of Network Edge Weights In Males

**eFigure 13.** Bootstrap Difference Test Of Network Edge Weights In Females

**eMethods**

**Assessment of Covariates**

In this study, several factors were considered as covariates for data analysis, including demographics (gender, age, body mass index (BMI), and place of residence), socioeconomic status (marital status and average monthly household income per capita), family structure (family type), health status (presence of chronic diseases), and lifestyle habits (smoking status, alcohol consumption, tea consumption, coffee consumption, sleep duration, and sleep quality). Marital status was categorized as "married" (including first marriage, remarriage, and married again) or "other" (including divorced, widowed, and unmarried). Family type was categorized as "core family" (parents and unmarried children), "couple family" (husband and wife only), or "other." "Other" family types included nuclear family (parents and married children), single-parent family (divorced, widowed, or unmarried parents and their children), single family (individuals who choose not to marry or remain single after divorce), DINK family (married couples who choose not to have children), skip-generation family (grandparents and grandchildren only, due to parental absence), extended family (parents and two or more married children or siblings living together), and other forms of family (stepfamilies, cohabiting families, same-sex families, etc.). Smoking status was defined as ever smoking (including conventional cigarettes, electronic cigarettes, or both). Alcohol consumption was defined as past drinking, current drinking, or always drinking. Finally, sleep quality was categorized as "good" (self-reported overall good or relatively good sleep) or "poor" (self-reported very poor or relatively poor sleep).

**eResults**

**Network accuracy and stability**

Stability testing of the network analysis yielded a CS=0.75, indicating that the network structure remains relatively unchanged even with the exclusion of 75% of the samples. As illustrated in eFigure 2, the correlation between the mean centrality measures of the sampled subsets and the centrality measures of the original sample gradually decreases as the sample size decreases, suggesting good stability of centrality measures. Bootstrap difference testing indicated statistical significance in the differences of node strength for most nodes (eFigure 3). Narrow bootstrap 95% confidence intervals substantiated the accurate estimation of edge weights in this study (eFigure 4).

**The comparison of network structures of different genders**

The comparison of network structures between genders revealed a significant influence of gender on smartphone use before bedtime and anxiety symptoms (Supplementary Figure 5). This influence is reflected in significant changes in the distribution of edge weights (M = 0.055; P = 0.007) and overall network strength (S = 0.033; P = 0.001) between the network models.

**Network accuracy and stability of different genders**

The stability test of network analysis showed a CS of 0.75 for both males (eFigure 8) and females (eFigure 9), indicating good stability of centrality indicators in both groups. Bootstrap difference tests revealed statistically significant differences in the strength of most nodes (eFigures 10 and 11). Furthermore, narrow bootstrap 95% confidence intervals in both male and female networks (eFigures 11 and 12) substantiated the accurate estimation of edge weights.

**eTable**

**eTable 1.** Linear Multiple Regression Analysis Of The Association Between Duration Of Smartphone Use Before Bedtime And The Severity Of Anxiety

| **Variables** | **B** | **SE** | **β** | **t** | **p** |
| --- | --- | --- | --- | --- | --- |
| Gender | -0.209 | 0.05 | -0.022 | -4.21 | ＜0.001 |
| Age | -0.003 | 0.002 | -0.01 | -1.317 | 0.188 |
| BMI | -0.213 | 0.059 | -0.018 | -3.623 | ＜0.001 |
| Residence | -0.31 | 0.053 | -0.029 | -5.815 | ＜0.001 |
| Marriage Status | 0.051 | 0.067 | 0.005 | 0.761 | 0.447 |
| Family Type | 0.244 | 0.029 | 0.044 | 8.285 | ＜0.001 |
| Suffering from chronic illness | 0.91 | 0.062 | 0.079 | 14.611 | ＜0.001 |
| Drinking | 0.31 | 0.057 | 0.03 | 5.473 | ＜0.001 |
| Smoking | 0.232 | 0.072 | 0.018 | 3.232 | 0.001 |
| Drinking tea | 0.101 | 0.049 | 0.011 | 2.071 | 0.038 |
| Drinking coffee | 0.37 | 0.056 | 0.034 | 6.601 | ＜0.001 |
| Length of sleep | 0.911 | 0.055 | 0.084 | 16.478 | ＜0.001 |
| Sleep quality | -2.003 | 0.065 | -0.159 | -30.83 | ＜0.001 |
| PIU Total Scores | 0.357 | 0.005 | 0.42 | 77.291 | ＜0.001 |
| Duration of Smartphone Use Before Bedtime | 0.116 | 0.056 | 0.011 | 2.065 | 0.039 |

adjusted R^2^=0.265, F=688.075 , P<0.001.

Abbreviations: BMI, Body Mass Index; PIU, problematic internet use.

**eTable 2.** Weighted Adjacency Matrix Of The Duration Of Smartphone Use Before Bedtime And Anxiety Symptoms

|  | GAD1 | GAD2 | GAD3 | GAD4 | GAD5 | GAD6 | GAD7 | phone |
| --- | --- | --- | --- | --- | --- | --- | --- | --- |
| GAD1 | 0.000 | 0.248 | 0.198 | 0.130 | 0.136 | 0.135 | 0.045 | 0.013 |
| GAD2 | 0.248 | 0.000 | 0.253 | 0.199 | 0.088 | 0.112 | 0.114 | 0.027 |
| GAD3 | 0.198 | 0.253 | 0.000 | 0.245 | 0.049 | 0.128 | 0.102 | 0.040 |
| GAD4 | 0.130 | 0.199 | 0.245 | 0.000 | 0.146 | 0.173 | 0.073 | 0.001 |
| GAD5 | 0.136 | 0.088 | 0.049 | 0.146 | 0.000 | 0.189 | 0.328 | -0.035 |
| GAD6 | 0.135 | 0.112 | 0.128 | 0.173 | 0.189 | 0.000 | 0.328 | 0.052 |
| GAD7 | 0.045 | 0.114 | 0.102 | 0.073 | 0.328 | 0.170 | 0.000 | 0.012 |
| phone | 0.013 | 0.027 | 0.040 | 0.001 | -0.035 | 0.052 | 0.012 | 0.000 |

**eTable 3.** Weighted Adjacency Matrix Of The Duration Of Smartphone Use Before Bedtime And Anxiety Symptoms In Males

|  | GAD1 | GAD2 | GAD3 | GAD4 | GAD5 | GAD6 | GAD7 | phone |
| --- | --- | --- | --- | --- | --- | --- | --- | --- |
| GAD1 | 0.000 | 0.211 | 0.196 | 0.111 | 0.165 | 0.139 | 0.077 | 0.000 |
| GAD2 | 0.211 | 0.000 | 0.234 | 0.198 | 0.113 | 0.125 | 0.124 | 0.015 |
| GAD3 | 0.196 | 0.234 | 0.000 | 0.247 | 0.064 | 0.100 | 0.120 | 0.032 |
| GAD4 | 0.111 | 0.198 | 0.247 | 0.000 | 0.132 | 0.185 | 0.066 | 0.009 |
| GAD5 | 0.165 | 0.113 | 0.064 | 0.132 | 0.000 | 0.208 | 0.289 | -0.007 |
| GAD6 | 0.139 | 0.125 | 0.100 | 0.185 | 0.208 | 0.000 | 0.000 | 0.037 |
| GAD7 | 0.077 | 0.124 | 0.120 | 0.066 | 0.289 | 0.000 | 0.000 | 0.024 |
| phone | 0.000 | 0.015 | 0.032 | 0.009 | -0.007 | 0.037 | 0.024 | 0.000 |

**eTable 4.** Weighted Adjacency Matrix Of The Duration Of Smartphone Use Before Bedtime And Anxiety Symptoms In Females

|  | GAD1 | GAD2 | GAD3 | GAD4 | GAD5 | GAD6 | GAD7 | phone |
| --- | --- | --- | --- | --- | --- | --- | --- | --- |
| GAD1 | 0.000 | 0.273 | 0.195 | 0.147 | 0.116 | 0.128 | 0.022 | 0.027 |
| GAD2 | 0.273 | 0.000 | 0.265 | 0.198 | 0.073 | 0.101 | 0.111 | 0.029 |
| GAD3 | 0.195 | 0.265 | 0.000 | 0.244 | 0.043 | 0.148 | 0.091 | 0.041 |
| GAD4 | 0.147 | 0.198 | 0.244 | 0.000 | 0.156 | 0.164 | 0.000 | 0.000 |
| GAD5 | 0.116 | 0.073 | 0.043 | 0.156 | 0.000 | 0.179 | 0.000 | -0.051 |
| GAD6 | 0.128 | 0.101 | 0.148 | 0.164 | 0.179 | 0.000 | 0.000 | 0.058 |
| GAD7 | 0.022 | 0.111 | 0.091 | 0.000 | 0.000 | 0.000 | 0.000 | 0.006 |
| phone | 0.027 | 0.029 | 0.041 | 0.000 | -0.051 | 0.058 | 0.006 | 0.000 |

**eFigure**


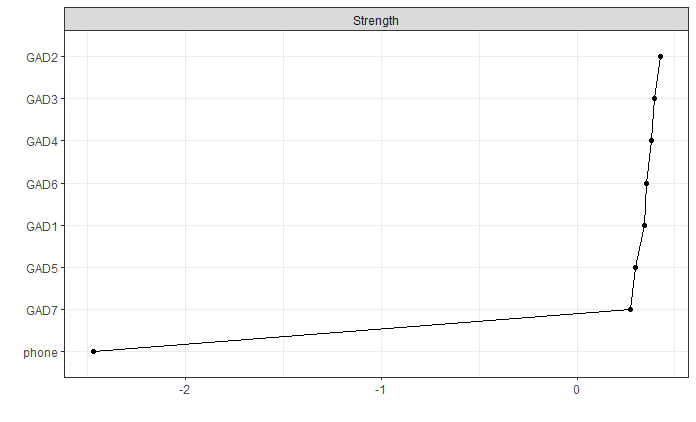


**eFigure 1.** Node Strength Centrality In The Network


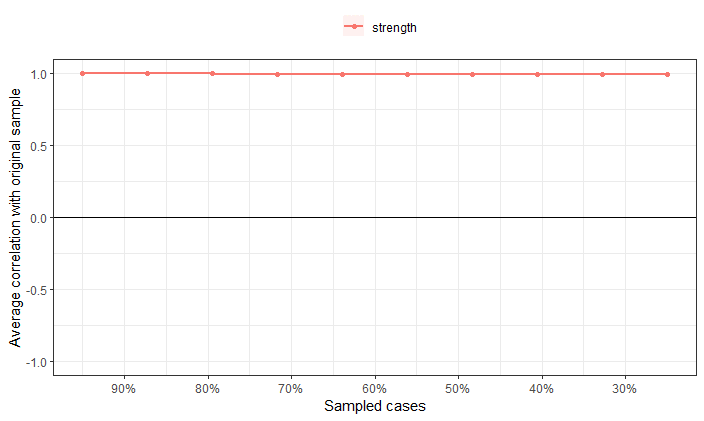


**eFigure 2.** Estimating The Stability Of The Network Structure Using The Case-Drop Subset Bootstrap Method

The X-axis represents the percentage of cases using the original sample. The Y-axis displays the mean correlation between the original network’s centrality index and the re-estimated network’s index.


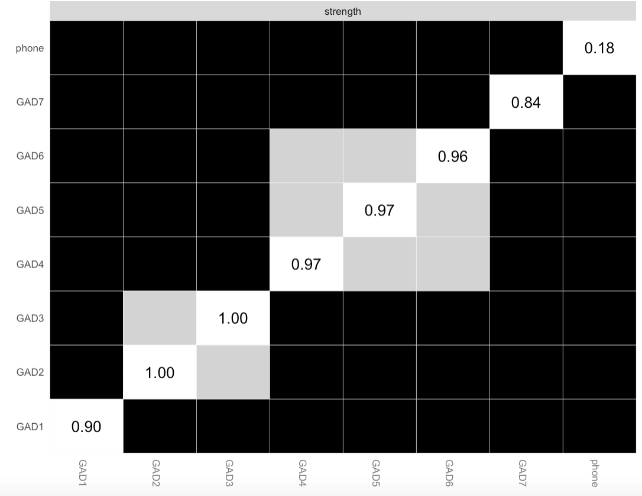


**eFigure 3.** Difference-In-Difference Test For Node Strength

This heatmap visualizes the results of the difference-in-difference test for node strength. Each square represents the comparison between two nodes. A black square indicates a statistically significant difference in strength between the corresponding nodes. Conversely, a gray square signifies no significant difference. The values within the white squares represent the strength difference between the nodes, with higher values indicating a larger difference.


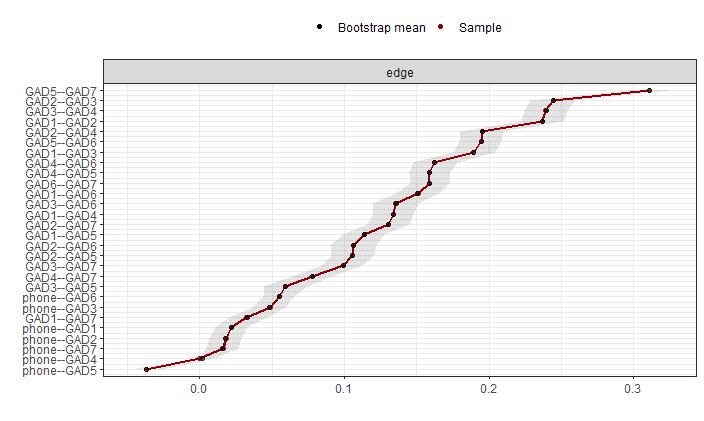


**eFigure 4.** Bootstrap Difference Test Of Network Edge Weights


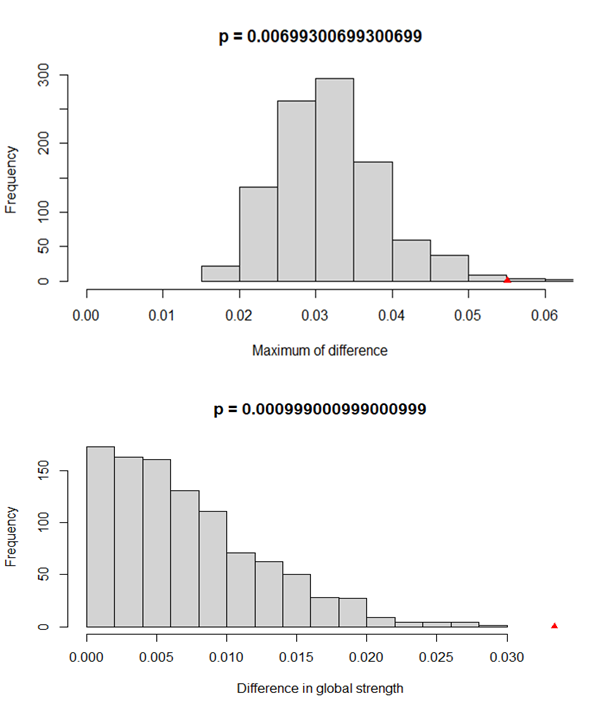


**eFigure 5.** Comparison Of Network Attributes Of Participants In Different Gender

Top Panel: Distribution of bootstrap values for the difference in network global strength between genders.

Bottom Panel: Distribution of bootstrap values for the maximum difference in any edge weight between genders (1000 permutations)


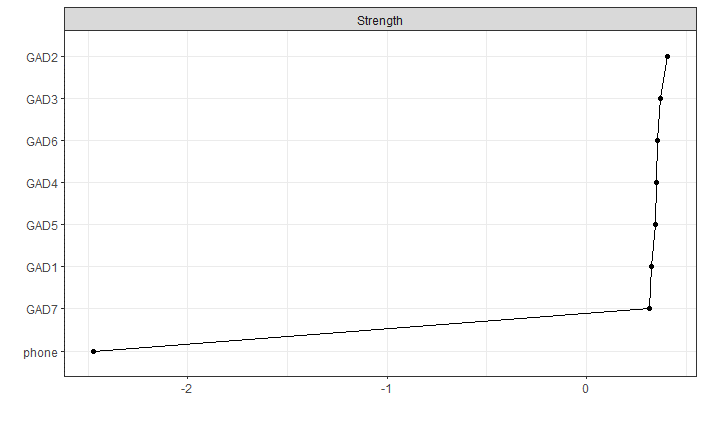


**eFigure 6.** Node Strength Centrality In The Network In Males


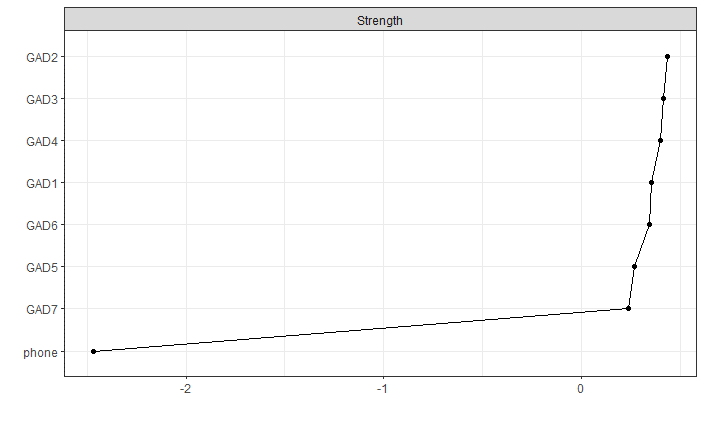


**eFigure 7.** Node Strength Centrality In The Network In Females


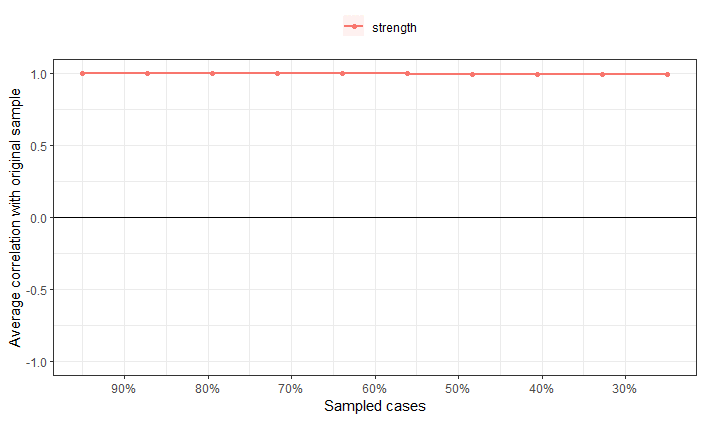


**eFigure 8.** Estimating The Stability Of The Network Structure Using The Case-Drop Subset Bootstrap Method In Males


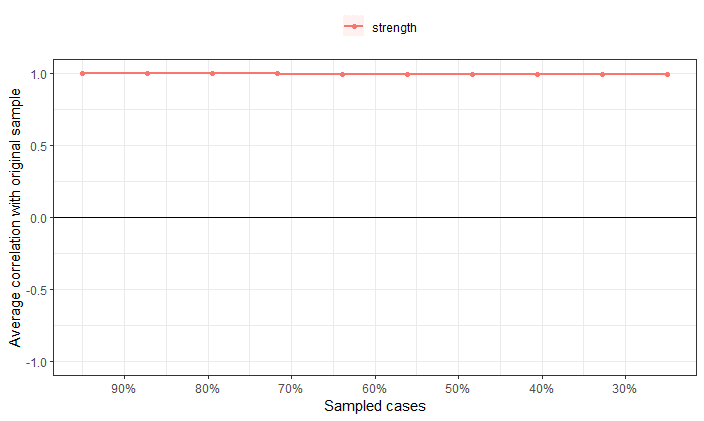


**eFigure 9.** Estimating The Stability Of The Network Structure Using The Case-Drop Subset Bootstrap Method In Fmales


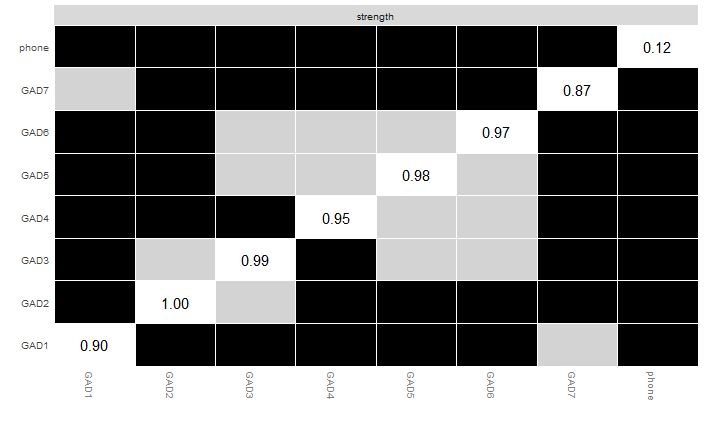


**eFigure 10.** Difference-In-Difference Test For Node Strength In Males


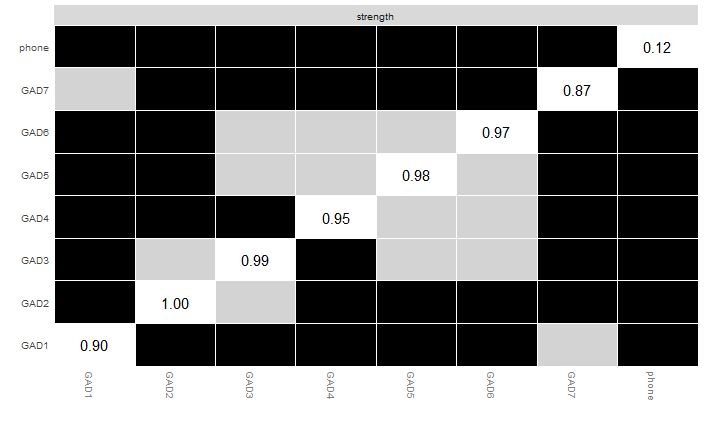


**eFigure 11.** Difference-In-Difference Test For Node Strength In Females


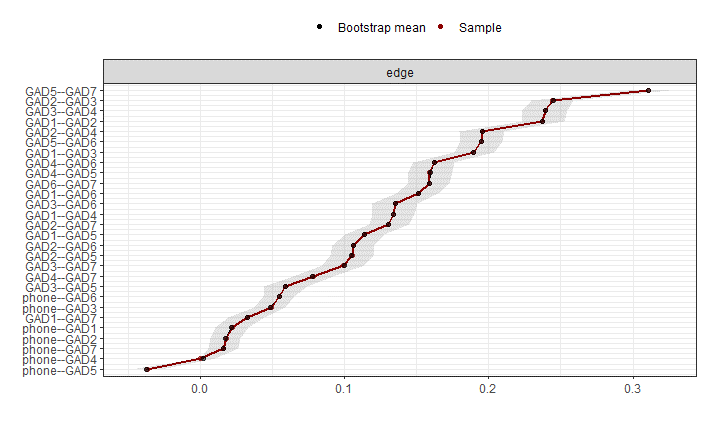


**eFigure 12.** Bootstrap Difference Test Of Network Edge Weights In Males


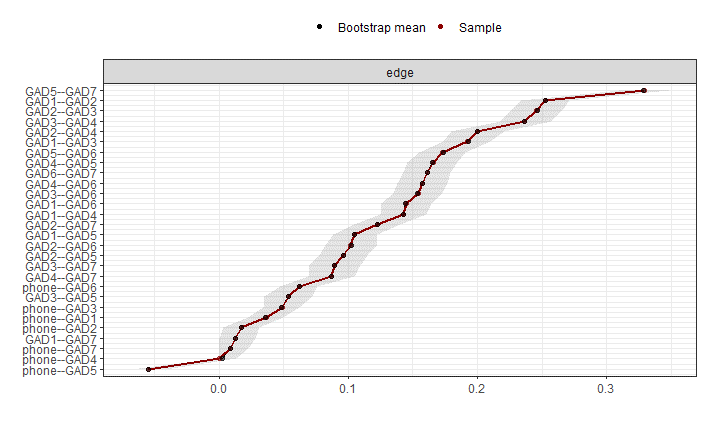


**e eFigure 13.** Bootstrap Difference Test Of Network Edge Weights In Females
